# A computer-based medical record system and personal digital assistants to assess and follow patients with respiratory tract infections visiting a rural Kenyan health centre

**DOI:** 10.1186/1472-6947-6-21

**Published:** 2006-04-10

**Authors:** Lameck Diero, Joseph K Rotich, John Bii, Burke W Mamlin, Robert M Einterz, Irene Z Kalamai, William M Tierney

**Affiliations:** 1 Moi University Faculty of Health Sciences, Eldoret, Kenya; 2 Indiana University School of Medicine, Indianapolis, IN, USA; 3 Regenstrief Institute, Inc., Indianapolis, IN, USA; 4 Mosoriot Rural Health Center, Nandi North District, Kenya

## Abstract

**Background:**

Clinical research can be facilitated by the use of informatics tools. We used an existing electronic medical record (EMR) system and personal data assistants (PDAs) to assess the characteristics and outcomes of patients with acute respiratory illnesses (ARIs) visiting a Kenyan rural health center.

**Methods:**

We modified the existing EMR to include details on patients with ARIs. The EMR database was then used to identify patients with ARIs who were prospectively followed up by a research assistant who rode a bicycle to patients' homes and entered data into a PDA.

**Results:**

A total of 2986 clinic visits for 2009 adult patients with respiratory infections were registered in the database between August 2002 and January 2005; 433 patients were selected for outcome assessments. These patients were followed up in the villages and assessed at 7 and 30 days later. Complete follow-up data were obtained on 381 patients (88%) and merged with data from the enrollment visit's electronic medical records and subsequent health center visits to assess duration of illness and complications. Symptoms improved at 7 and 30 days, but a substantial minority of patients had persistent symptoms. Eleven percent of patients sought additional care for their respiratory infection.

**Conclusion:**

EMRs and PDA are useful tools for performing prospective clinical research in resource constrained developing countries.

## Background

The potential of health care information technology to support clinical and research activities has not been fully explored in developing countries. Proper assessment of the processes and outcomes of care is required before resources can be appropriately allocated and care interventions can be planned. Having reliable clinical data from community health centers would therefore be invaluable in making decisions and planning interventions.

Although electronic medical records are becoming increasingly prevalent in developed countries, they are still rare in developing countries. Using electronic medical records (EMRs) and personal digital assistants (PDAs) to identify patients for prospective investigations and collect outcomes data can increase the efficiency of such investigations. The feasibility of using PDAs for longitudinal data collection in poor rural settings has not been fully explored. Their portability and long-lasting batteries support structured data collection in a variety of geographic locations and may prove more useful for data collection in the field than laptop or tablet computers, especially in remote areas and in developing countries where electricity may not be available. However, their small screens, propensity to lose data when their batteries die, and lack of a wide range of programming tools can be disadvantages for using PDAs for field research.

In this article, we describe how we used an existing EMR in a rural Kenyan primary care center was used along with PDAs to assess the care and outcomes of one of the most prevalent conditions at the center: acute respiratory tract infections.

## Methods

This study was approved by Indiana University Institutional Review Board and the Institutional Research Ethics Committee of Moi University College of Heath Sciences.

### The Mosoriot Medical Record System

The Mosoriot Medical Record System (MMRS) was installed in February 2001 as a collaborative project between Indiana University and Moi University [1-3]. The overall aim of developing this system was to improve health care delivery and as a clinical and public health research tool. Details of the basic design and implementation of the system are described elsewhere [1-3]. In brief the MMRS is comprised of a paper encounter form-data template, data entry module, report generating module, and a data dictionary. The system runs on two IBM-compatible computers linked by a cross over network cable. One computer is used for check in and registration and the other used for check out and entering encounter data. The core MMRS utilizes a Microsoft Access^® ^relational database that consists of four tables, namely the registration table, visit table, drug/laboratory test results table, and data dictionary. The registration table contains one record per patient, the fields in this table include patient identification number, name, village, and date of the registration visit. The visit table contains a record per visit (identified uniquely by patient identifier number and visit date), the clinic(s) visited, diagnoses, services provided, plus the amount of money paid. The drug/lab table contains records for each drug prescribed and results of each diagnostic test performed. The data dictionary contains a record for each data elements with descriptors and, where appropriate, limits on data entry. Only coded and numeric data, as defined by the data dictionary, are entered into the MMRS.

On arrival at the health center patients are given paper encounter forms upon which health center staff record patient data. The patient carries this form to the health center's various clinics, laboratories, pharmacy, and financial office. At the end of the visit, the data from this form are keyed into the MMRS checkout computer.

An assessment of the impact of MMRS on the workflow at Mosoriot rural health centre was done by performing formal time motion studies before and after implementation [3]. After MMRS implementation, patient visits were 22% shorter, they spent 58% less time with providers and 38% less time waiting. The MMRS reports have also facilitated detection of clustering of sexually transmitted diseases in one village and lack of immunization in another village and this lead to a team of health personnel being dispatched to the villages to carry out appropriate interventions [3].

### Respiratory tract infections in Kenya

Respiratory tract infections account for 20% of hospital admissions and 25% of deaths in Kenyan hospitals [4,5]. Acute respiratory infections are also responsible for a large number of cases of absence from work, school, and home activities. People living in rural areas can be characterized as vulnerable populations, predisposed to poor health outcomes because of lack of sanitation (potable water, sewage and garbage disposal) and limited access to preventive health care [6]. Lower respiratory tract infections such as bronchopneumonia and bronchitis occur significantly more often in rural areas [7]. There is increased mortality associated with presentation to rural health facilities [8]. Community verbal autopsy in one rural area in Kenya (Muranga) found that 45% of deaths in children were related to acute respiratory tract infections [9]. Respiratory tract infections are also important causes of morbidity and mortality in HIV-infected patients [10]. In addition, one study in Kenya demonstrated that the prevalence of respiratory tract infections is directly related to poor dwellings [11]. Higher mortality in adults admitted to hospital was also reported in patients who were unemployed, visited traditional healers, and had no education, characteristics which are all more prevalent in rural areas [11].

Improving the quality of medical treatment in rural communities is a priority. Despite a high incidence of acute respiratory tract infections in Kenya, information on clinical correlates with acute illnesses, treatment outcomes, and costs of care are lacking. Although there is limited information available concerning children cared for in rural [12] and urban clinics [13], there is only one published study that assesses the prevalence of respiratory infections in adults [14] which was community-based and did not represent the experience of rural health care providers. Moreover, there are insufficient data on the prevalence of various comorbid conditions and health seeking behaviors among Kenyan patients with acute respiratory infections or factors correlated with various outcomes. Poor documentation in medical notes and lack of local data may compromise good clinical practice. Longitudinal data collection among patients with acute respiratory infections could help identify high risk patients for adverse outcomes who might benefit from more intensive treatment at their initial presentation.

### The Mosoriot Rural Health Centre

The health center is situated in North Rift Valley Province of Kenya. It provides health care to the surrounding population of more than 40,000 people. It is also a rural training center for traditional birth attendants and community health workers as well as medical students, clinical officers, and nurses. Services offered include diagnosis and treatment of acute illnesses in children and adults, some basic laboratory services (urinalysis, blood smears for malaria, sputum microscopy for tuberculosis, HIV antibody testing, blood glucose concentration, etc), antenatal and well-child care, family planning, and treatment of sexually transmitted diseases.

Mosoriot is a public health training site for the Moi University Faculty of Health Sciences' medical students who spend 25% of their time on field attachments doing community-based public health research. During the attachments the students learn how a community is organized, its resources, the health problems, and the delivery and management of health services. The health problems of the community identified by the students are then linked with MMRS data available at the local health center. This is important in planning community interventions.

The aim of this project was to provide data for needs assessment, designing intervention studies, and generating hypotheses for research for patients with acute respiratory tract infections. Specifically, we used MMRS data and data collected into a personal data assistant (PDA) from patients at the Mosoriot Rural Health Center and later in their homes.

### Modifications of the MMRS

Implementing the prospective system for assessing acute respiratory infections required modifying the MMRS, programming the PDA data entry system, and training the health center staff and research assistant in their use. The focus of this project was patients who had clinical features of acute respiratory tract infections. A case was defined by an adult seeking care for illness with one or more of the following symptoms: cough, shortness of breath, sputum production, chest pains, hemoptysis, fever, chills, running nose, or wheezing.

**Figure 1:**
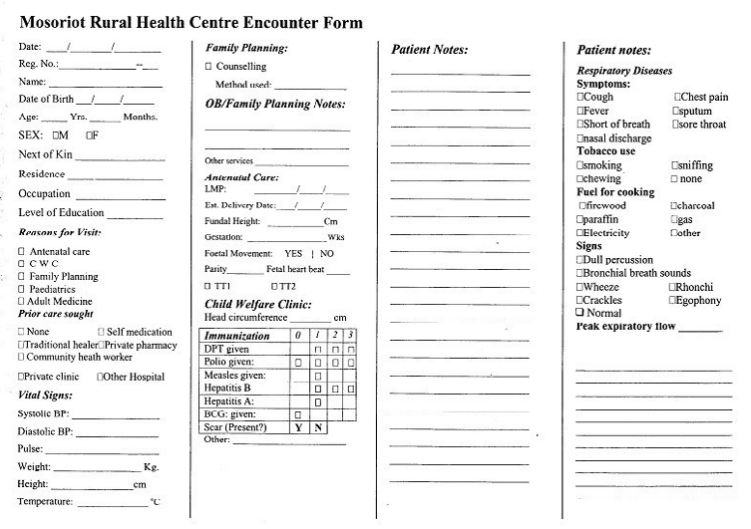
MMRS encounter form modified to collect data on acute respiratory illness. This section begins near the bottom of column 3 and carries over to column 4.

**Figure 2:**
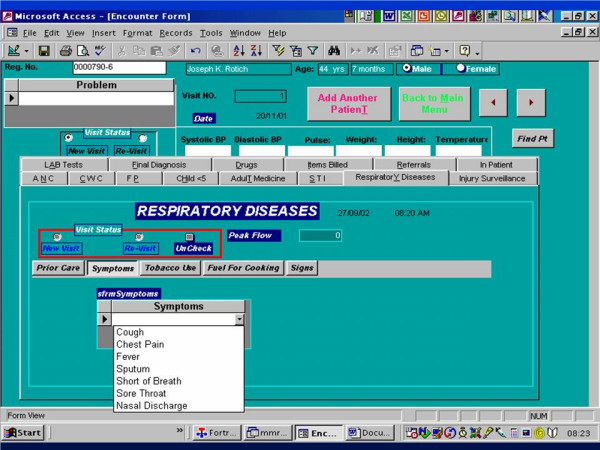
MMRS data entry screen used to enter data on acute respiratory illness.

The paper MMRS encounter form was modified to capture data specific to acute respiratory tract infection (Figure 1). These were added as check boxes and included; prior care sought, such as visits to traditional healers, private pharmacies, shops, private clinics and other health institutions; historical elements such as cough, chest pains, fever, sputum production, shortness of breath, and hemoptysis; tobacco use and type of fuel used for cooking; physical signs such as wheezing, bronchial breathing, and crepitations; and chest X-ray results. In most cases, the examining clinician obtained and recorded a peak expiratory flow rate. These changes were integrated into the paper encounter forms and into the MMRS data entry module, the latter by adding checkboxes and dropdown lists on prior care sought, tobacco use, symptoms, physical signs, and laboratory results (Figure 2).

We implemented changes in the MMRS in three stages: installing the new encounter forms and MMRS upgrades; training the clinicians to record the patient data on the encounter form and the checkout clerk to enter them into the MMRS; and performing pilot tests of the modified system. The training was provided to five clinical officers, four records clerks, and ten nurses and emphasized the new fields added to the encounter form and MMRS data entry module. For the success of such systems the cooperation and acceptance of the health center personnel is important. Therefore, their input was sought in the content and redesign of the encounter form and MMRS data entry module.

The next stage in the implementation was to assess the functional and technical performance of the system. This was done by allowing the staff to use the new encounter forms and then keying the new data elements into the computer. From the data they entered we generated summaries and reports specifically for patients with acute respiratory infections. The health center staff assessed the usefulness of the reports and summaries produced and provided additional input on their design and implementation.

**Figure 3:**
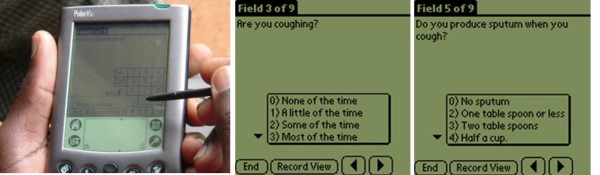
The programmed PDA and sample screens. A: PDA in use. B: Screen used to collect data on cough. C: Screen used to collect data on sputum production.

The questionnaires for assessing treatment outcomes were then programmed using Pendragon Forms7 into Palm V7 PDAs. The data entry fields were restricted to either check boxes or fields where only appropriate data could be entered. Appropriate skip patterns were also programmed into the PDAs to remove errors in navigating the questionnaires. The programmed PDA and sample screens are shown in Figure 3. The research assistants were trained on the use of PDAs to administer questionnaires. The symptom-related questions were adopted from the bronchitis and chronic obstructive airways disease symptoms questionnaire created by the Pneumonia Patient Outcome Research Team [15,16]. The symptom-related questions included presence and severity of cough, sore throat, shortness of breath, sputum production, chest pains, fatigue, and fever. The questions on work related activities and medication compliance were adapted from the Short Form-36 health survey [17].

### Subject enrollment

Review of MMRS data before the study showed that approximately 20 patients with acute respiratory illnesses presented to the Mosoriot health centre every day. For logistic reasons, we felt that the research assistant enrolling patients could enroll a maximum of three patients per day. These three patients recruited per day were chosen using systematic sampling whereby every fifth patient assigned a visit diagnosis of acute respiratory infection was approached and invited to participate in the study. This was done until three patients were recruited for a particular day, and then recruitment ended for that day.

### Data collection

**Figure 4:**
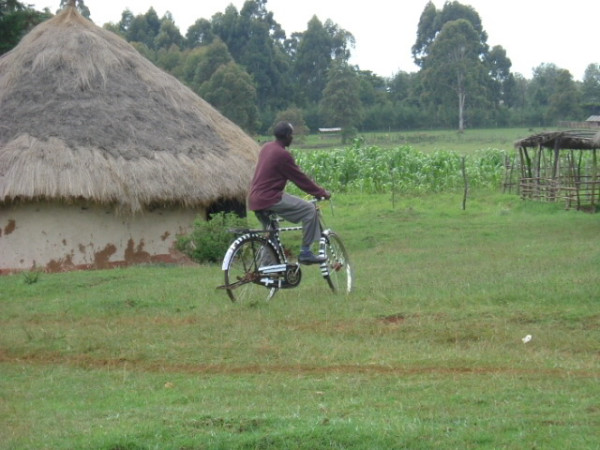
Research assistant arriving in a patient's village on a bicycle.

**Figure 5:**
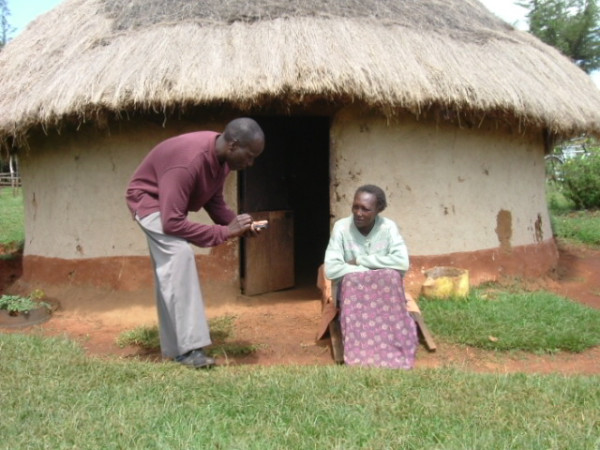
Research assistant collecting outcome data on a subject.

A research assistant used the PDA-based structured questionnaire to assess enrolled patients in the health center (following the clinical visit) on Day 1 and in the patients' homes on Days 7 and 30. Research assistants used bicycles as their primary means of transport to visit patients in their homes (Figures 4 and 5). During the follow-up visits the outcomes that were assessed included resolution of symptoms specific to acute respiratory tract infections, drugs taken, hospitalization, and time away from work or usual activities. At the end of each week, each research assistant uploaded data (by either infrared beaming of data or use of a cradle) from the PDA into a Microsoft Access database that was also created on a desktop microcomputer using Pendragon Forms. These data were then merged with data from the MMRS.

## Results

### Subjects enrolled and their characteristics

Between August of 2002 and January 2005, there were 2986 visits to the health center made by 2009 patients for acute respiratory illnesses. The majority of the visits were due to upper respiratory tract infection (49%) followed by pneumonia and tonsillitis. The distribution of clinical diagnoses made by the health centre providers is shown in Table 1. Of these, 433 agreed to be followed up in their homes and signed informed consent statements in either English, Kiswahili, or Kalenjin (the local tribal language). Of the 433 patients enrolled, 60% were females with a mean age of 32 years; only 6% of the patients were more than 60 years of age.

**Table 1:** Diagnosis distribution.

**Diagnosis**	**Number (%)**
Upper respiratory tract infections	1449 (49%)
Pneumonia	463 (16%)
Asthma	297 (10%)
Tonsillitis	286 (10%)
Bronchitis	195 (6.5%)
Bronchospasm	71 (2.4%)
Tuberculosis	58 (1.9%)
Bronchopneumonia	32 (1.1%)
Otitis Media	29 (1.0%)
Rhinitis	26 (0.9%)
Pharyngitis	19 (0.6%)
Sinusitis	7 (0.2%)
Otitis externa	4 (0.1%)

### Clinical outcomes of respiratory illnesses

During follow up 52 patients (12%) could not be located in their homes. There were 4 deaths. All the deaths occurred at home. Complete merged data were available for all enrolled subjects receiving follow-up interviews.

Table 2 shows the proportion of patients reporting symptoms during the follow up. Chest pains (77%) and cough (73%) were the most commonly reported symptoms on the day of enrollment. All symptoms reported by the patients showed a steady decline in prevalence with subsequent follow ups. Despite there being significant improvement in symptoms at 7 and 30 days, a substantial minority of patients had persistent symptoms 30 days after their presentation to the health center. At day 30 chest pains and cough were still more often reported compared to the other symptoms. Overall 85% of the patients felt subjectively better by day 30 with only 11% reporting seeking further care

**Table 2:** Proportion of patients reporting symptoms during follow up.

**Symptom**	**Day 1**	**Day 7**	**Day 30**
Chest pains	77%	61%	60%
Cough	73%	64%	51%
Sputum production	66%	48%	37%
Feeling tired	62%	52%	31%
Fever	62%	34%	24%
Shortness of breath	53%	38%	31%
Sore Throat	42%	27%	23%
Felt better		81%	85%
Sought further medical care		17%	11%

## Discussion

We previously established that computer based medical records is feasible in a developing country [3]. This study demonstrates that such an EMR and hand-held computing devices such as PDAs can be effectively used even in resource-constrained countries to perform prospective clinical research. Minor modifications were necessary to the paper encounter form and data entry program that were easily adapted to by health center clinical and administrative staff. The PDA was easily portable even on a bicycle whereas paper forms would have been bulky and difficult to manage. The screen was easy to see, and data entry via stylus was efficient. The questionnaire's skip patterns were easily navigated, and missing data were eliminated by not allowing the research assistant to proceed in the questionnaire without completing the necessary data fields. Entry of erroneous or inconsistent data was prevented. The cost of the PDA was more than offset by not having to pay data entry technicians to enter data from paper questionnaires into a computer database. Data entry errors were also avoided. Others have shown that the total time for data collection on a handheld device and downloading to a PC was 23% faster and had 58% fewer errors than hand recording data on paper forms [18]. Although loss of data following loss of battery power was always possible, it did not occur during this study. On the downside, the data entry program did not allow for entry of text field notes by the research assistants, who had to use a paper notebook for such notes. This could have caused a disconnect between these text notes (which were uncommon) and the patient data to which they referred.

When implementing electronic medical record systems, provision should be made for future expansion and modifications. This should take into account the rapid changes in clinical practice, disease patterns, and specific research needs. Feedback from staff and their suggestions should be considered when redesigning and expanding the database [19]. In our case, discussions were held with the staff, including the research assistants gathering data at the health centre and in the field, to identify some of the priority health problems in the surrounding communities that needed adequate data capture with possible plans for intervention. This was done by amending existing MMRS encounter forms and data entry screens and adding a disease-specific section to the encounter form and adding a tab and a sub-screen that contained fields relevant to acute respiratory diseases. The health center clinical and administrative staff aided in the identification of appropriate data and formatting of the paper and electronic data recording interfaces. This helped reduce fears and distrust of computers [20] and engaged the clinical staff in the clinical research project.

## Conclusion

Even simple EMRs such as the MMRS, which gather data for clinical purposes during individual patient visits, can be used to enhance longitudinal care and disease management, both at the level of the individual patient through the health center and even to higher levels of health system management. In addition, PDAs can be effective tools for capturing primary outcome data and linking them to the data collected during initial health center visits for selected illnesses and conditions. This study should encourage others that simple EMRs and PDAs can be used to perform relevant clinical research even in severely resource-constrained environments.

## Abbreviations

EMR: electronic medical record

PDA: personal data assistant

ARI: acute respiratory illness

MMRS: Mosoriot Medical Record System

## Competing interests

The author(s) declare that they have no competing interests.

## Authors' contributions

Made substantial contributions to the conception, design, acquisition of data, or analysis and interpretation of results: Diero, Rotich, Bii, Mamlin, Einterz, Kalamai, Tierney.

Involved in drafting, revising, or critiquing the manuscript: Diero, Tierney.

Given final approval to the manuscript version submitted: Diero, Rotich, Bii, Mamlin, Einterz, Kalamai, Tierney.

## Pre-publication history

The pre-publication history for this paper can be accessed here:


